# Correction: Insight into mineralizer modified and tailored scorodite crystal characteristics and leachability for arsenic-rich smelter wastewater stabilization

**DOI:** 10.1039/d4ra90100b

**Published:** 2024-09-20

**Authors:** Yonggang Sun, Qi Yao, Xin Zhang, Hongling Yang, Na Li, Zhongshen Zhang, Zhengping Hao

**Affiliations:** a Department of Environmental Nano-materials, Research Center for Eco-Environmental Sciences, Chinese Academy of Sciences Beijing 100085 P. R. China zhangxin@rcees.ac.cn +86-10-62843096 +86-10-62843688; b National Engineering Laboratory for VOCs Pollution Control Material & Technology, University of Chinese Academy of Sciences Beijing 101408 P. R. China

## Abstract

Correction for ‘Insight into mineralizer modified and tailored scorodite crystal characteristics and leachability for arsenic-rich smelter wastewater stabilization’ by Yonggang Sun *et al.*, *RSC Adv.*, 2018, **8**, 19560–19569, DOI: https://doi.org/10.1039/C8RA01721B.

The authors regret that an incorrect version of **Fig. 11a** was included in the original article. The correct version of **Fig. 11a** is presented here.
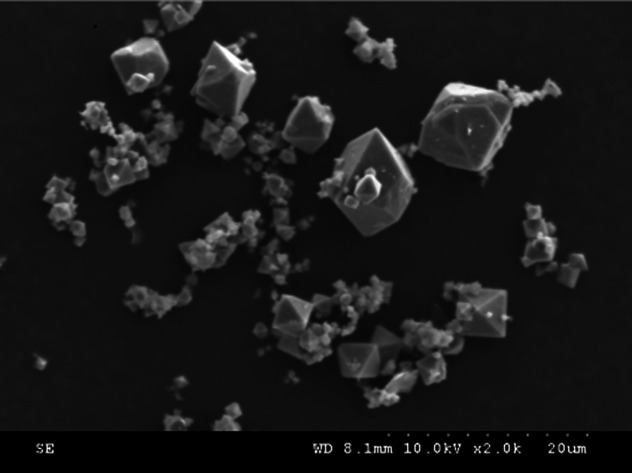


**Fig. 11**(a) SEM image for solid precipitate at 120 °C – H_2_O control group.

The Royal Society of Chemistry apologises for these errors and any consequent inconvenience to authors and readers.

